# Screening Commercially Available Entomopathogenic Biocontrol Agents for the Control of *Aethina tumida* (Coleoptera: Nitidulidae) in the UK

**DOI:** 10.3390/insects3030719

**Published:** 2012-08-09

**Authors:** Andrew G. S. Cuthbertson, James J. Mathers, Lisa F. Blackburn, Michelle E. Powell, Gay Marris, Stephane Pietravalle, Mike A. Brown, Giles E. Budge

**Affiliations:** The Food and Environment Research Agency, National Bee Unit, Sand Hutton, York YO41 1LZ, UK; james.mathers@fera.gsi.gov.uk (J.J.M.); lisa.blackburn@fera.gsi.gov.uk (L.F.B.); michelle.powell@fera.gsi.gov.uk (M.E.P.); gay.marris@fera.gsi.gov.uk (G.M.); stephane.pietravalle@fera.gsi.gov.uk (S.P.); mike.brown@fera.gsi.gov.uk (M.A.B.); giles.budge@fera.gsi.gov.uk (G.E.B.)

**Keywords:** *Aethina tumida*, *Apis mellifera*, biological control, entomopathogenic agents

## Abstract

The Small hive beetle, *Aethina tumida*, is an invasive pest of honey bees. Indigenous to sub-Saharan Africa, it has now become established in North America and Australia. It represents a serious threat to European honey bees. Commercially available entomopathogenic agents were screened for their potential to control beetle larvae. Entomopathogenic fungi investigated had minimal impact. The nematodes *Steinernema kraussei* and *S. carpocapsae* provided excellent control with 100% mortality of larvae being obtained. Sequential applications of the nematodes following larvae entering sand to pupate also provided excellent control for up to 3 weeks. The information gained supports the development of contingency plans to deal with *A. tumida* should it occur in the UK, and is relevant to the management of Small hive beetle where it is already present.

## 1. Introduction

In its native range the Small hive beetle (*Aethina tumida* Murray, Coleoptera: Nitidulidae) (SHB) is an occasional parasite and scavenger of honey bee colonies indigenous to sub-Saharan Africa [1,2]. However, as an invasive species it has caused much economic damage, and since 1996 has become established in North America and Australia [2,3,4]. It has also recently been discovered in Hawaii. The SHB has yet to be reported in Europe, South America or Asia. 

The SHB lifecycle consists of a pupation stage that occurs outside the beehive in the surrounding soil. Depending on temperature this life-stage can last for varying periods of time. For example, at temperatures ranging between 20–30 °C, Cuthbertson *et al*. [5] showed adult beetles to begin emerging from soil following 18 days and continuing up until 84 days. Small hive beetle development is known to be slower at lower temperatures [6]. Both larvae and pupae can be found in the soil. Therefore, there is an opportunity for control measures to be applied at this stage that will not have any impact upon the bees in the hive. Beekeepers have traditionally used pesticides containing permethrin to control larvae and pupae in the soil [7], however continued use of this can give rise to resistance [8] and undesirable side effects on both honey bees and humans [9,10]. Therefore, there is much demand to improve the range of products available for the control of the larvae and pupae stages. Such alternative control agents include entomopathogenic nemadodes (EPN) and fungi (EPF), which have successfully been used against other invertebrate pests [11,12,13]. In regards to *A. tumida*, the infectivity of three species of nematodes towards wandering larvae (the larval stage that is actively seeking a pupation site) was shown to be moderate [12]. This study aimed to screen species of entomopathogenic agents that are commercially available in the UK for their potential to be used against Small hive beetle.

## 2. Results and Discussion

**Figure 1:**
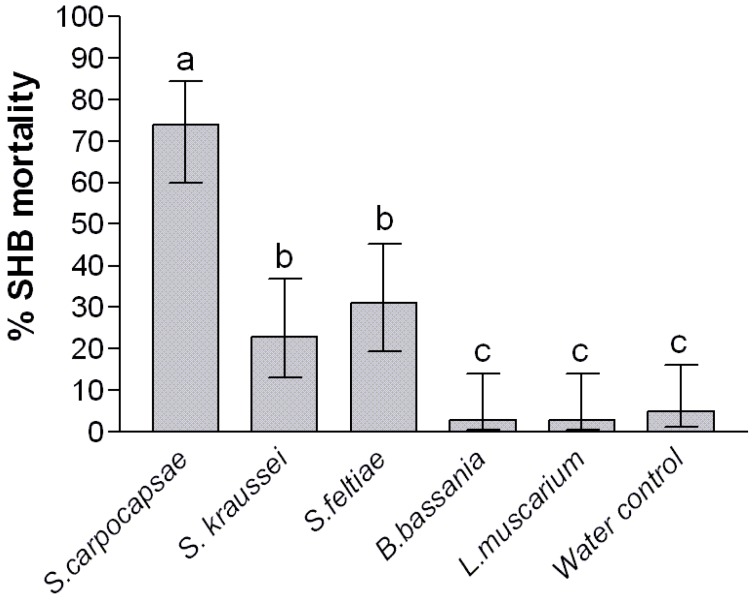
Impact of direct exposure of control agents on *Aethina tumida* wandering larvae after 2 weeks. Error bars represent the 95% confidence intervals. Means were separated using least significant differences after adjusting for multiple comparisons. Significantly different means (5% significant level) are represented by suffixes a–c.

**Figure 2:**
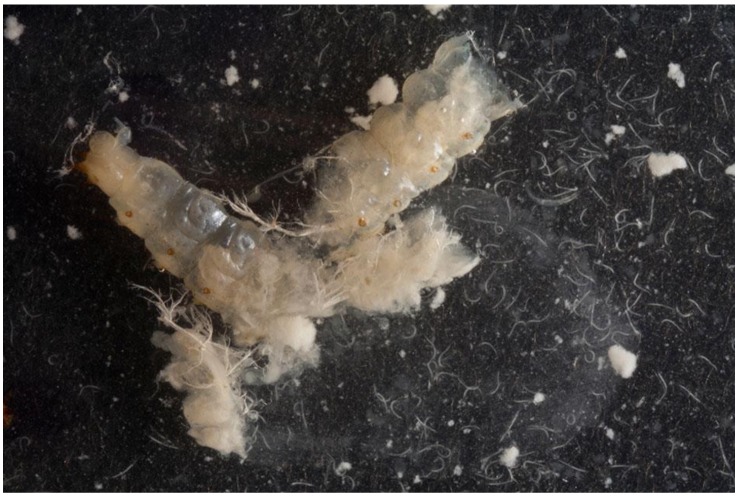
Dissected *Aethina tumida* larvae releasing the entomopathogenic nematode *Steinernema carpocapsae*.

Direct exposure demonstrated a significant treatment effect on the wandering larvae when compared to the control (*p *< 0.001), however neither entomopathogenic fungus caused mortality above that seen in the water controls (Figure 1). The entomopathogenic nematodes showed more promise; *S. carpocapsae* achieved significantly higher mortality than *S. kraussei* and *S. feltiae* (*p *< 0.05), which in turn achieved significantly higher mortality than the control after 2 weeks. Upon dissecting the larvae, nematodes freely emerged from the body cavity confirming their ability to infect the larvae (Figure 2). It has been stated that susceptibility of insects to control agents generally declines with increasing insect size. This has been demonstrated with mermithid nematodes against mosquito larvae [14]. However, as nematodes enter through the natural openings of the larvae, Gaugler and Molloy [15] showed that susceptibility was a function of larval size with larger larvae being more susceptible to nematode infection, simply due to the fact that it was easier for nematodes to enter the natural openings.

**Figure 3:**
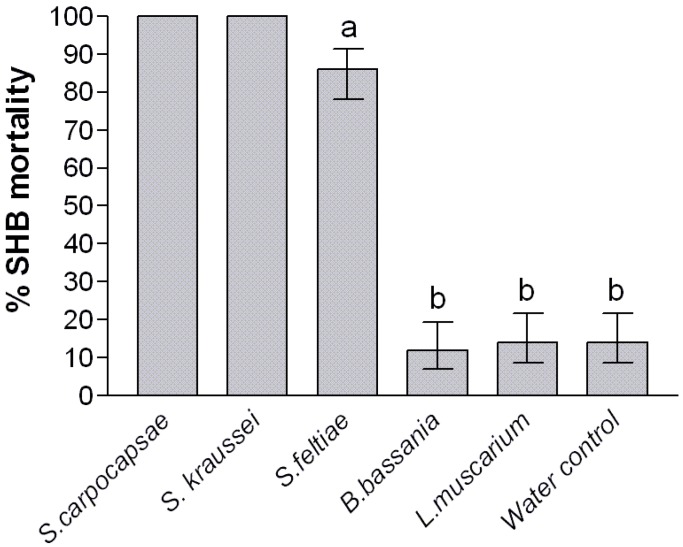
Impact of in-direct exposure of control agents on *Aethina tumida* wandering larvae. Error bars represent the 95% confidence intervals. Means were separated using least significant differences after adjusting for multiple comparisons. Significantly different means (5% significant level) are represented by suffixes a and b.

Treating the sand before adding the larvae exposes the SHB to the biocontrol agents during pupation, and more closely simulates how beekeepers might apply such products in the field. Indirect exposure demonstrated a significant treatment effect on SHB mortality when compared to the control (*p *< 0.001), however, once again neither entomopathogenic fungus caused mortality above that seen in the water controls (Figure 3). Treating the sand produced excellent results for *S. kraussei* and *S. carpocapsae* where total mortality of *A. tumida* was achieved. No adults emerged from either of these two treatments and neither did sieving the sand yield any remains of developing beetles, suggesting the larvae became infected, died and their remains dissolved during the 6 week test. Also, *S. feltiae* achieved significantly higher mortality than the control (*p *< 0.05; Figure 3).

**Figure 4:**
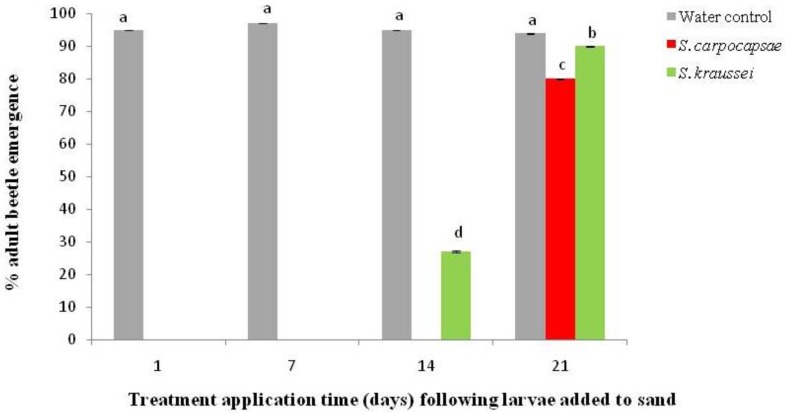
Impact of sequential applications of entompathogenic nematodes on *Aethina tumida* wandering larvae following their submergence in sand. Beetle emergence assessed after 6 weeks following larvae being added to sand. Error bars represent the 95% confidence intervals. Means were separated using least significant differences after adjusting for multiple comparisons. Significantly different means (5% significant level) are represented by suffixes a–d.

Following sequential application of the nematodes, significant reduction in adult beetle emergence was obtained (*p *< 0.001; Figure 4) for up to 3 weeks following larvae entering the sand to pupate. Under laboratory conditions the nematodes were deemed viable in the sand following 1 week after application. Nematodes were observed under a light microscope moving between the sand grains [16].

The data from our screening trials are consistent with those of Cabanillas and Elzen [12] and Ellis *et al*., [17] who demonstrated that *A. tumida* larvae and pupae are susceptible to entomopathogenic nematodes. In our study nematode efficacy varied with nematode species. In general the nematodes are known to penetrate the host and feed/reproduce for approximately 14 days [18]. After this period new infective juveniles emerge from the host and begin to seek a new host. Nematodes are thought to be better at locating hosts when they are dispersing from a host, showing evidence of a programming process in regards to host preference [19]. Therefore, it would be suggested that as long as *A. tumida* pupae are available as a food source, any applied inoculant nematode population would be more likely to be self-sustaining. However, any break in host availability may demand the soil be retreated with nematodes. 

The fungi in our study had limited effect. Reasons for this are unclear. The fungal genera *Beauveria* and *Lecanicillium *are generalist entomopathogens, with species and strain-dependent differences in specificity and virulence against a range of insect groups [20]. They also have a cosmopolitan distribution and can be isolated from insects, mites, soil and a variety of other substrates [21]. Therefore, *Beauveria* and *Lecanicillium* would seem ideal candidates for the control of *A. tumida*. Temperature and humidity are important factors in fungi growth and germination. The ground surrounding beehives can vary greatly in moisture content. Our laboratory studies aimed to mimic conditions as closely as possible as to what is found surrounding beehives. Muerrle *et al*. [22] obtained promising mortality of *A. tumida* using *B. bassiana. *Ellis *et al*. [23] suggested that the fungi *Aspergillus flavus* and *A. niger* can cause mortality of larvae and pupae of *A. tumida*. However, these two fungi are known to cause mortality of larval and adult honey bees [24]. Therefore, further investigations would require the testing of more species and individual strains of fungi that have the potential to be commercially available in the UK for the control of *A. tumida*.

## 3. Experimental Section

### 3.1. Insect Rearing and Control Agents

*Aethina tumida* were cultured and maintained as described by Cuthbertson *et al*. [5] under strict quarantine conditions. Final instar (wandering) larvae were used for all experimental trials. The control agents investigated are all commercially available products in the UK and across Europe and comprised 3 EPN’s: *Steinernema feltiae* (Nemasys), *S. kraussei *(NemasysL), *S. carpocapsae* (Capsanem) and 2 EPF’s: *Lecanicillium muscarium* (Mycotal) and *Beauveria bassiana *(Naturalis). The impact of direct and in-direct exposure along with sequential application of the agents showing most potential were investigated in separate experiments.

### 3.2. Direct Exposure of Larvae to Control Agents

For direct exposure trials, individual wandering larvae were dipped in recommended dose rates of the fungi (10^8^ conidia/mL) and nematode products (10,000 infective juveniles/mL) for 3 seconds. They were then placed on moist filter paper within 9 cm diameter Petri dishes and maintained at 20 °C, 65% R.H. and 16:8 h light:dark regime. Ten larvae were placed in each dish with 10 dishes per treatment product. Larvae dipped in water and placed on moist filter paper in Petri dishes acted as controls. Mortality for all treatments was assessed after 2 weeks.

### 3.3. Indirect Exposure of Larvae to Control Agents

For indirect exposure, 7 cm diameter by 15 cm tall plastic containers were filled with sand (8% moisture content). 50 mL of control product (500,000 nematode IJ or 50^9^ fungal conidia) was added over the surface of the sand at the same dose rates as in the direct trials. Once the solution had soaked down into the sand, ten wandering larvae were added to the surface. The containers were then sealed and maintained at the conditions described above. There were ten containers per treatment. Controls consisted of wandering larvae added to containers in which the sand had been treated with 50 mL of water. Treatments were maintained for 6 weeks in order to allow adult beetles to emerge. Mortality was calculated as the number of beetles that failed to emerge. In order to confirm the fate of those individuals that did not emerge as adult beetles, at the end of each trial the sand substrate was sieved and searched for insect debris.

### 3.4. Sequential Application of Nematodes against Beetle Larvae

For sequential application trials using the two nematode species (*S. carpocapsae* and *S. kraussei*), containers as described above were prepared. Batches of ten wandering SHB larvae were added to each container. Following 24 hours the first batch of nematode solution was added to 10 containers. Then at weekly intervals, nematode treatments were added (at the same dose rate as before; 50 mL of product (500,000 IJ’s)) to separate batches of the original larvae infested containers. There were 10 replicates per nematode treatment per application date (four application dates in total). Control containers received an equal volume (50 mL) of water. Following treatment all containers were maintained in a CE room (23 °C, 65% r.h.) for 6 weeks to allow beetles ample opportunity to emerge. Nematode controls (sand pots with no beetle larvae present) were also maintained to determine their longevity in the sand.

### 3.5. Analysis of Data

The data was analysed using a Generalized Linear Model (GLM) with a binomial distribution and logit link function. 95% confidence intervals were calculated on the logit scale, then back-transformed (as proportions) and the treatments were grouped, following pairwise testing (at the 5% significance level) using means separating groups on the logit scale.

## 4. Conclusions

In conclusion, our trials demonstrate that commercially available entomopathogenic nematodes in the UK can infest and kill *A. tumida* wandering larvae. Furthermore, these products are available across Europe, and so have the potential to be used as control agents should the Small hive beetle expand its range to this continent. Further work is now required to determine economic dose rates and time spans between applications to ensure full control of *A. tumida*. The information gathered all supports the development of contingency plans to deal with *A. tumida* should it ever be located within the UK [25].
